# Effects of a Serious Smartphone Game on Nursing Students' Theoretical Knowledge and Practical Skills in Adult Basic Life Support: Randomized Wait List–Controlled Trial

**DOI:** 10.2196/56037

**Published:** 2024-04-05

**Authors:** Nino Fijačko, Ruth Masterson Creber, Špela Metličar, Matej Strnad, Robert Greif, Gregor Štiglic, Pavel Skok

**Affiliations:** 1 Faculty of Health Sciences University of Maribor Maribor Slovenia; 2 Maribor University Medical Centre Maribor Slovenia; 3 School of Nursing Columbia University New York, NY United States; 4 Medical Dispatch Centre Maribor University Clinical Centre Ljubljana Ljubljana Slovenia; 5 Faculty of Medicine University of Maribor Maribor Slovenia; 6 Community Healthcare Center Dr Adolfa Drolca Maribor Maribor Slovenia; 7 European Resuscitation Council Research Net Niels Belgium; 8 University of Bern Bern Switzerland; 9 School of Medicine Sigmund Freud University Vienna Vienna Austria; 10 Faculty of Electrical Engineering and Computer Science University of Maribor Maribor Slovenia

**Keywords:** serious smartphone game, adult basic life support, teaching, game, games, gaming, education, nurse, nursing, nurses, educational, mHealth, mobile health, app, apps, application, applications, smartphone, smartphones, RCT, randomized controlled trial, controlled trial, technology-enhanced learning, TEL, life support, knowledge retention, theoretical knowledge, practice, practical, resuscitation

## Abstract

**Background:**

Retention of adult basic life support (BLS) knowledge and skills after professional training declines over time. To combat this, the European Resuscitation Council and the American Heart Association recommend shorter, more frequent BLS sessions. Emphasizing technology-enhanced learning, such as mobile learning, aims to increase out-of-hospital cardiac arrest (OHCA) survival and is becoming more integral in nursing education.

**Objective:**

The aim of this study was to investigate whether playing a serious smartphone game called MOBICPR at home can improve and retain nursing students’ theoretical knowledge of and practical skills in adult BLS.

**Methods:**

This study used a randomized wait list–controlled design. Nursing students were randomly assigned in a 1:1 ratio to either a MOBICPR intervention group (MOBICPR-IG) or a wait-list control group (WL-CG), where the latter received the MOBICPR game 2 weeks after the MOBICPR-IG. The aim of the MOBICPR game is to engage participants in using smartphone gestures (eg, tapping) and actions (eg, talking) to perform evidence-based adult BLS on a virtual patient with OHCA. The participants’ theoretical knowledge of adult BLS was assessed using a questionnaire, while their practical skills were evaluated on cardiopulmonary resuscitation quality parameters using a manikin and a checklist.

**Results:**

In total, 43 nursing students participated in the study, 22 (51%) in MOBICPR-IG and 21 (49%) in WL-CG. There were differences between the MOBICPR-IG and the WL-CG in theoretical knowledge (*P*=.04) but not in practical skills (*P*=.45) after MOBICPR game playing at home. No difference was noted in the retention of participants’ theoretical knowledge and practical skills of adult BLS after a 2-week break from playing the MOBICPR game (*P*=.13). Key observations included challenges in response checks with a face-down manikin and a general neglect of safety protocols when using an automated external defibrillator.

**Conclusions:**

Playing the MOBICPR game at home has the greatest impact on improving the theoretical knowledge of adult BLS in nursing students but not their practical skills. Our findings underscore the importance of integrating diverse scenarios into adult BLS training.

**Trial Registration:**

ClinicalTrials.gov (NCT05784675); https://clinicaltrials.gov/study/NCT05784675

## Introduction

Sudden cardiac arrest is one of the leading causes of death in adults worldwide. It is responsible for over a million deaths annually [1]. Most deaths occur in the out-of-hospital setting, and the outcome possibly can be improved with proper adult basic life support (BLS) [2]. Effective implementation of adult BLS can double the chances of survival after a sudden cardiac arrest [3,4]. Reviews report poor cardiopulmonary resuscitation (CPR) by nursing students, despite the completion of adult BLS certification [5]. BLS knowledge and skills decline significantly within months of initial training [5,6]. For this reason, the European Resuscitation Council (ERC) and American Heart Association guidelines recommend shorter and more frequent adult BLS training as it helps retain adult BLS content longer and maintain competency levels [7,8]. Currently, adult BLS education in higher nursing education institutions traditionally imparts theoretical knowledge through a frontal approach and teaches practical skills using manikins and automated external defibrillators (AEDs), although the approach can vary significantly from one university to another [5,9].

A noticeable generational shift is evident in health care systems, both in Europe and abroad, characterized by the increasingly common employment of younger individuals. These younger future health care employees bring a higher proficiency in technology and information literacy [10,11], attributes cultivated from growing up in an era dominated by modern technology [12]. Technology-enhanced learning (TEL) approaches, developed to improve adult BLS knowledge and skill retention, ultimately aim to increase out-of-hospital cardiac arrest (OHCA) survival [8]. The most recent adult BLS guidelines highlight the integration of TEL into adult BLS courses [8,13,14]. This includes not only immersive technologies, such as extended reality [15], but also mobile learning (m-learning), which has increased dramatically in nursing education in recent years [16]. A recent meta-analysis indicates that serious smartphone games are a promising and effective tool for adult BLS education [17].

M-learning, by its definition, encompasses the use of mobile technology [18], with mobile apps on smartphones often serving as the educational platform [19]. Research has demonstrated m-learning’s beneficial effects on fostering a variety of learning outcomes and competencies in the field of nursing [20,21]. Smartphone-based m-learning [21] seamlessly complements education through serious games and gamification [15]. Gamification involves applying game design elements to nongame contexts [22], such as educational content in higher education [23]. Conversely, serious games are crafted to use a specific type of game (eg, computer or mobile games) for the purpose of learning about significant subjects, such as adult BLS content education at the higher education level [24].

To the best of our knowledge, only a limited number of studies have explored the use of serious smartphone games for teaching adult BLS to health care students [25-29]. Among these, only 1 study demonstrated an improvement in both the theoretical knowledge and practical skills associated with adult BLS [28]. Other studies have reported enhancements in either theoretical knowledge [29] or practical skills related to adult BLS. The positive effects of a serious smartphone game can be seen as early as 2 weeks [25,26], as well as 1 month after the intervention [27-29]. Studies have compared different teaching methods, where the use of serious smartphone games seems to have better results than simulation-based learning but is less effective than virtual reality–based game learning [26,30]. Some studies have also shown improvements in practical skills, such as compression rate accuracy [27,28], although this tends to be inferior when compared to simulation-based methods [30]. In contrast, in 2 studies, serious smartphone games did not provide notable benefits and led to worse performance in theoretical and practical areas, although students showed a clear preference in favor of serious smartphone games [27,28].

The aim of the study was to evaluate whether playing a serious smartphone game called MOBICPR [31] at home can enhance nursing students’ theoretical knowledge of and practical skills in adult BLS.

## Methods

### Study Protocol

The study was conducted at the Faculty of Health Sciences, University of Maribor (Maribor, Slovenia) between March and May 2023. The study was registered in ClinicalTrials.gov (NCT05784675). The study protocol was written in accordance with the Consolidated Standards of Reporting Trials of Electronic and Mobile Health Applications and Online Telehealth (Multimedia Appendix 1) [32].

### Ethical Considerations

Ethical approval was obtained from the Slovenian National Medical Ethics Committee (0120-157/2018), and permission to conduct the study on the faculty premises was obtained from the Faculty of Health Sciences, University of Maribor. During an oral presentation of the study, nursing students were informed about the research protocol, and written consent was obtained afterward. Data confidentiality and anonymity were maintained throughout the study. Participants were rewarded for their participation in the study with a free beverage from a vending machine and a copy of the *Game Changer* painting by street artist Banksy [33].

### Participants

All nursing students enrolled in the first-degree nursing program at the Faculty of Health Sciences, University of Maribor, during the 2022-2023 academic year were invited to participate in the study. Inclusion criteria to participate in the study were written informed consent, an age of at least 18 years, and ability to perform adult BLS on a manikin (eg, without injury). Our study had no exclusion criteria.

### Study Design and Randomization

This study had a randomized wait list–controlled design, where nursing students were randomly assigned in a 1:1 ratio using a computer-generated list (Microsoft 365 Excel Enterprise) to either a MOBICPR intervention group (MOBICPR-IG) or a wait-list control group (WL-CG). The WL-CG was a group of nursing students who were assigned to a wait list and received the intervention (MOBICPR game for playing at home) 2 weeks after the MOBICPR-IG.

### Interventions

All assessments of the participants’ theoretical knowledge of and practical skills in adult BLS were conducted 3 time points: baseline, 2-week follow-up, and 4-week follow-up. At the baseline assessment, the investigators first collected demographic data from the participants. Additionally, the participants were questioned about their willingness to assist both family members and strangers during OHCA with CPR. Prior to practical skills in adult BLS, participants’ the theoretical knowledge of adult BLS was assessed using a questionnaire with 33 single- and multiple-choice questions [25,34-36] on an open source online survey app called 1ka (Ljubljana, Slovenia); see Multimedia Appendix 2. A back-translation approach was used for translating the questionnaire into the Slovenian language.

**Figure 1:**
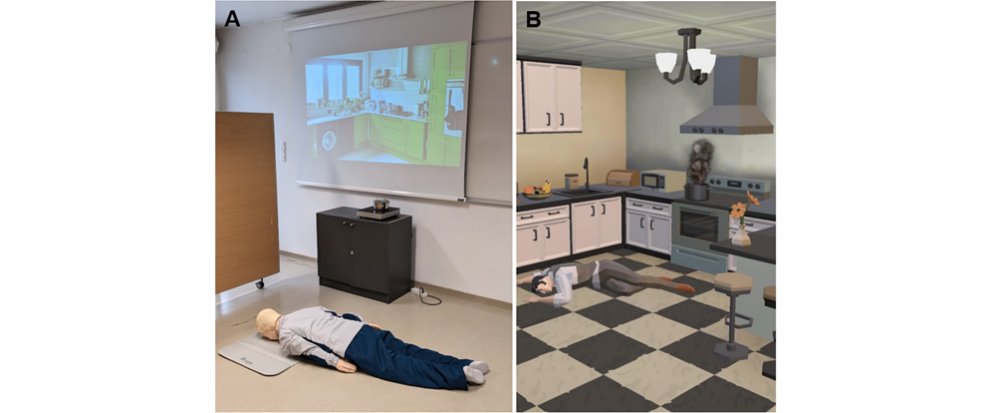
Staged kitchen with the Resusci Anne QCPR in the middle (A) and a cartoon person in the MOBICPR game lying on the floor in a kitchen (B). QCPR: Quality Cardiopulmonary Resuscitation.

Prior to the assessment of adult BLS practical skills, each participant was given a scenario based on OHCA to read (Multimedia Appendix 3). After reading the scenario, the participants were given a smartphone for calling emergency services at the time of performing adult BLS. Instead of dialing the actual emergency number, the participants used the contact stored on the smartphone as 112 (ie, the Slovenian emergency number). After the call was placed by each participant, the investigator answered the phone and conducted a simulated dispatcher conversation [37]. The investigator was a registered nurse working at the local medical dispatch center. Each participant performed 2-minute adult BLS without any help in a staged kitchen on a manikin (Resusci Anne Quality Cardiopulmonary Resuscitation [QCPR], Laerdal Medical) using an AED (Defibtech, Trainer AED). The staged kitchen was a space surrounded by mobile walls in the hospital’s simulated room. A photo of a kitchen was projected onto the wall, and below it was an electric stove with a pot full of water (Figure 1). In each adult BLS scenario, the investigators turned on the electric stove, and the scenario began when the water started to boil, simulating a hazard. The kitchen was chosen because the majority of OHCAs occur there [38].

After 2 minutes of performing adult BLS, each participant received assistance from an outside person bringing in an AED and taking over CPR. The adult BLS practical skills of each participant were recorded using a Sony Handycam 4K AX53 camera and an Apple iPad Pro 3rd generation tablet. Two investigators with a background in emergency medicine and teaching laypersons adult BLS assessed the participants’ practical skills in adult BLS using a modified checklist [25,36,39] according to the ERC BLS guidelines of 2015 [40] and 2021 [34], with a total of 34 items (Multimedia Appendix 4). A back-translation approach was used for translating the checklist into the Slovenian language. Numerical data from the SkillReporter for Tablet version 1.4.1 (Laerdal Medical) app installed on a Samsung Galaxy Tab S6 Lite tablet was also included in the evaluation of the participants’ practical skills in adult BLS. Investigator debriefing was not conducted following the assessment of the participants’ adult BLS theoretical knowledge and practical skills. Instead, each participant (from MOBICPR-IG at baseline and from WL-CG 2 weeks after baseline) first played the MOBICPR game [31] on a Samsung Galaxy A13 smartphone in front of the investigator and then received the same smartphone to play at home. The objective of the MOBICPR game is for participants to interact with a smartphone using gestures (eg, tapping) and actions (eg, talking) to help save the life of a virtual patient with OHCA by performing evidence-based adult BLS. The MOBICPR game is based on the 2021 ERC BLS guidelines [34], and the BLS content was developed using the Delphi process. The patient’s chance of survival in the MOBICPR game reduced with each incorrect interaction by the participants. At the end of the MOBICPR game, each participant received a total score in the form of a gamification feature that corresponded to the risk of survival (score>50% meant the patient survived) [41]. Gamification, defined as “using game design elements in non-game contexts,” has been introduced into nursing education to promote engagement using features such as leaderboards, rewards, badges, and avatars [22]. After playing the MOBICPR game as much as they wanted for 2 weeks, participants in the MOBICPR-IG returned the smartphones. Participants in the W-CG then received the smartphones and followed the same protocol as participants in the MOBICPR-IG, that is, they played the MOBICPR game in front of the investigator before taking the smartphone home. Participants in the W-CG also returned the smartphones after playing the MOBICPR game at home for 2 weeks. Additionally, at the study’s conclusion, each participant was asked an open-ended question regarding the number of family members or friends with whom they shared the MOBICPR game for playing.

### Outcome Measures

The primary outcomes were (1) assessment of the participants’ theoretical knowledge of adult BLS using a questionnaire with a total maximum score of 33 points, where each correct answer was awarded 1 point (Multimedia Appendix 2), and (2) assessment of the participants’ practical skills in adult BLS using a checklist with a total maximum score of 39 points (Multimedia Appendix 4).

The secondary outcome was a summary score of high-quality CPR components: (1) a chest compression (CC) rate of 100-120 beats per minute (bpm), (2) a CC depth of 50-60 mm, (3) CC fraction>80%, and (4) a rescue breath volume of 500-600 mL (Multimedia Appendix 4). All measures were taken as mentioned earlier [16,23,27,28]. A total QCPR score was also included, ranging from 0% to 100%. More detailed information about software scoring is available on the Laerdal Medical website [42]. Both primary and secondary outcomes were measured at 3 time points: baseline, 2-week follow-up, and 4-week follow-up.

### Statistical Analysis

Statistical analyses were conducted in October and November 2023. Data were analyzed using the R statistical programming language (R Foundation for Statistical Computing). The data presented in the summary table were prepared using frequency analysis, which also included a chi-square test to assess the similarity of the distribution between the intervention and control groups. Theoretical knowledge and practical skill assessments were averaged at the item level and subsequently analyzed using nonparametric statistical tests (Wilcoxon paired-sample test and Mann-Whitney *U* test) as the normality of the distribution was violated. As nonnormal distribution might represent a problem when calculating mean values, violin plots were also used for the purpose of visualizing aggregated scores due to their ability to visualize the distribution of the data. *P*<.05 was considered statistically significant. Effect size (η^2^) values >0.1 represented a small effect; 0.3, a moderate effect; and ≥0.5, a large effect. Continuous variables were analyzed according to the Gaussian distribution and reported as the mean (SD) or the median (IQR), whichever was appropriate.

## Results

### Participant Details

**Figure 2:**
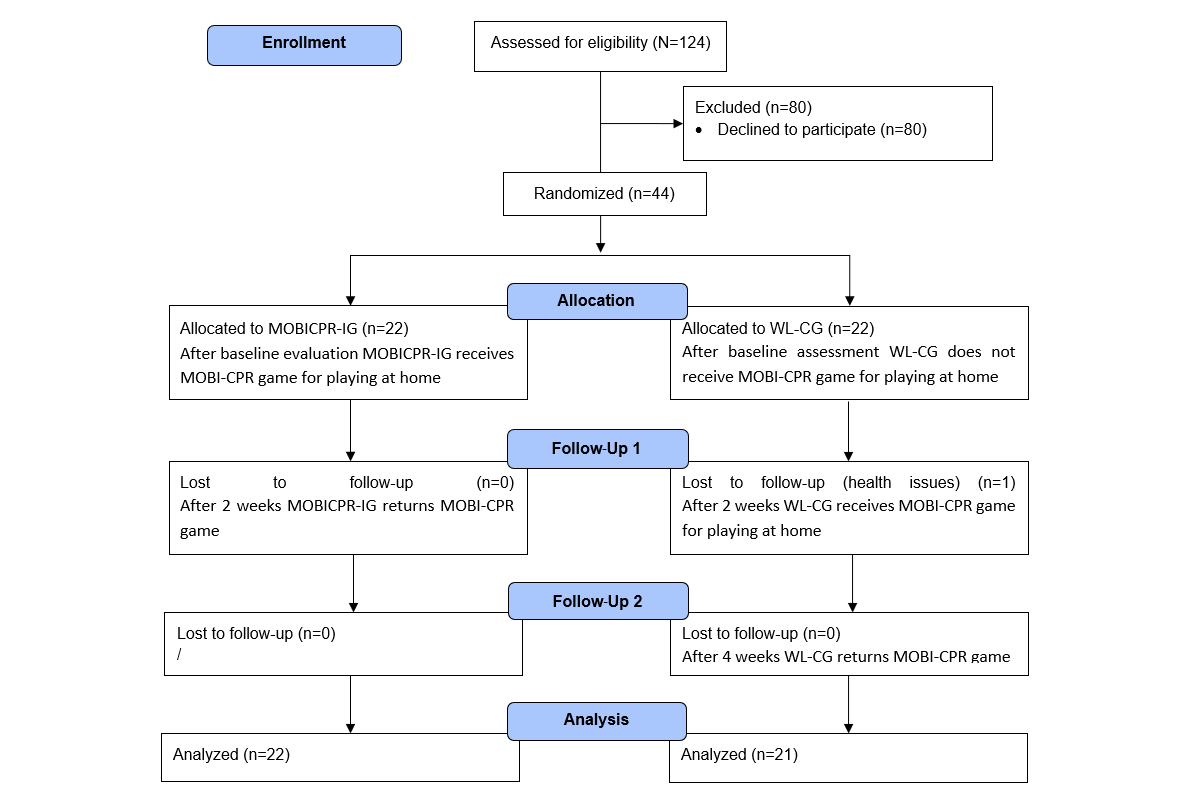
Flow diagram of study participants. MOBICPR-IG: MOBICPR intervention group; WL-CG: wait-list control group.

Of 124 nursing students, 80 (64.5%) declined to participate in the study and 44 (35.5%) were enrolled into the study. At follow-up, 1 (5%) of the 22 participants in the WL-CG dropped out. In the end, 43 (98%) of 44 participants were included in the final analysis (Figure 2).

The mean age of the participants was 19 (SD 0.6) years, 38 (88%) were female, 35 (81%) had a background in health care and nursing education, 32 (74%) had an iOS smartphone, and the self-reported mean daily time spent on the smartphone was 3.8 (SD 1.2) hours (Table 1).

**Table 1:** Baseline demographic characteristics.^a^

Characteristics		Total participants (N=43), n (%)	MOBICPR-IG^b^ (n=22), n (%)	WL-CG^c^ (n=21), n (%)	*P* value	
**Gender**						.67
	Male	5 (12)	3 (14)	2 (10)	—^d^	
	Female	38 (88)	19 (86)	19 (90)	—	
**Age (years)**						.90
	19	30 (70)	16 (73)	14 (67)	—	
	20	11 (26)	5 (23)	6 (29)	—	
	21	2 (5)	1 (5)	1 (5)	—	
**Education**						.19
	Health care and nursing	35 (81)	18 (82)	17 (81)	—	
	High school/gymnasium	5 (12)	4 (18)	1 (5)	—	
	Pharmacy	2 (5)	0	2 (10)	—	
	Economy	1 (2)	0	1 (5)	—	
**Operating system on smartphone**						.001
	Apple iOS	32 (74)	11 (50)	21 (100)	—	
	Android	11 (26)	11 (50)	0	—	

^a^The percentages may exceed 100 because of rounding.

^b^MOBICPR-IG: MOBICPR intervention group.

^c^WL-CG: wait-list control group.

^d^Not applicable.

All participants had received some previous adult BLS training. However, only 2 (5%) had witnessed a cardiac arrest. Most of them (n=38, 88%) had already performed CCs on manikins, but only a few had also been giving rescue breaths (n=12, 28%) and used any kind of AED (n=13, 30%). All participants (n=43, 100%) expressed a willingness to assist a patient with OCHA and perform adult BLS. In addition, they all expressed a willingness to perform mouth-to-mouth resuscitation on a family member or acquaintance. However, only about half of them (n=19, 44%) were willing to do the same for a stranger. The predominant concern for not administering rescue breaths to unknown individuals was the uncertainty regarding the patient’s medical history and the risk for infectious diseases, as cited by 22 (92%) of the 24 (56%) participants who expressed reluctance. On average, each participant introduced and shared the MOBICPR game with 3 (SD 2) family members or friends for trial and play.

### Primary Outcomes

To assess the differences between the 2 groups at all 3 observed time points, we calculated the cumulative scores of adult BLS theoretical knowledge and practical skills for both groups.

**Figure 3:**
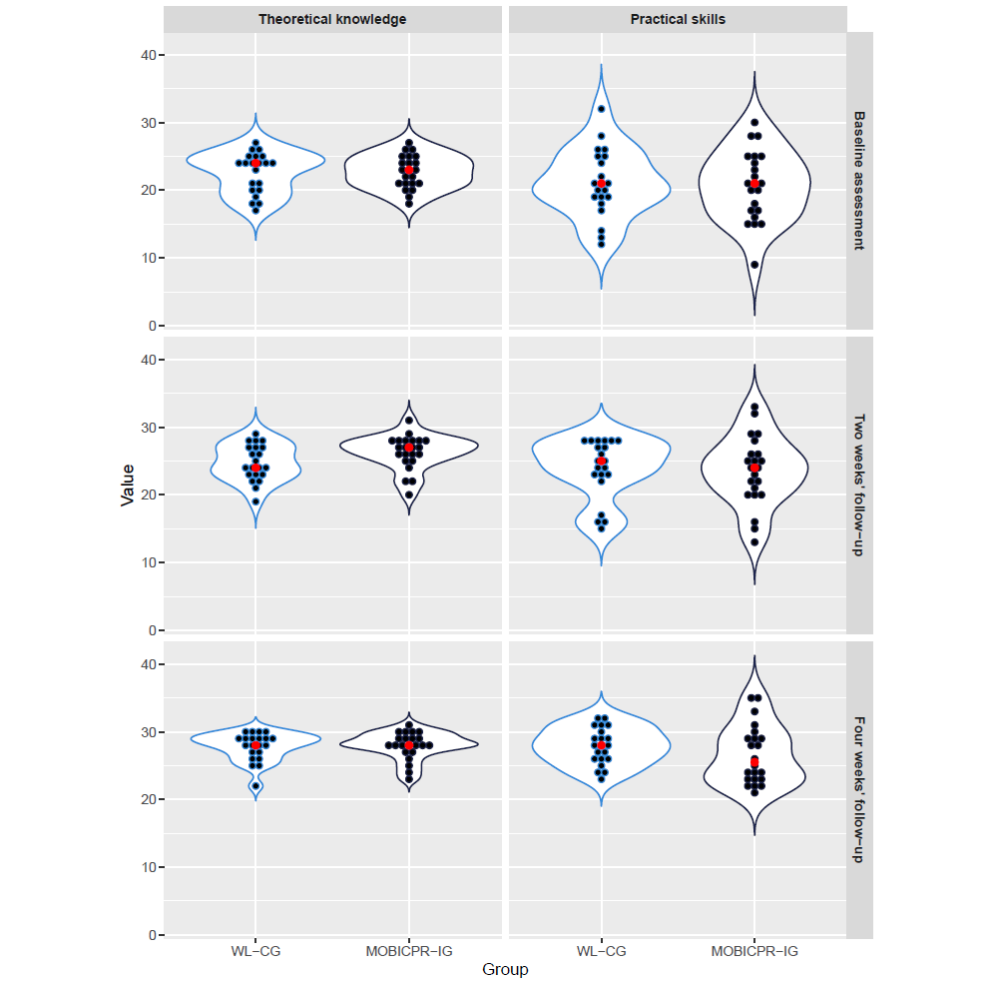
Comparison of theoretical knowledge and practical skill scores for all study participants at baseline, 2-week follow-up, and 4-week follow-up measurements. MOBICPR-IG: MOBICPR intervention group; WL-CG: wait-list control group.

Figure 3 shows that playing the MOBICPR game at home for 2 weeks improved the overall adult BLS theoretical knowledge (median gain of 4 points, IQR 3, η^2^=0.113, *P*=.005) and practical skills (median gain of 4 points, IQR 7, η^2^=0.05, *P*=.04). However, in the WL-CG, which waited for 2 weeks to play the MOBICPR game at home, the theoretical knowledge of adult BLS improved by 2 points (IQR 4, η^2^=0.302, *P*=.001), whereas the practical skills in adult BLS increased by 3 points (IQR 3, η^2^=0.018, *P*=.14). In the MOBICPR-IG, after 2 weeks of not playing the MOBICPR game at home, the retention of theoretical knowledge gained an additional 2 points (IQR 2, η^2^=0.019, *P*=.13) and practical skills gained 3 points (IQR 3.75, η^2^=0.122, *P*=.003) compared to the 2-week follow-up.

To focus on the impact of playing the MOBICPR game on adult BLS theoretical knowledge and practical skills, we observed participants in both groups and calculated the difference in the cumulative points for both groups after they played the MOBICPR game at home for 2 weeks.

**Figure 4:**
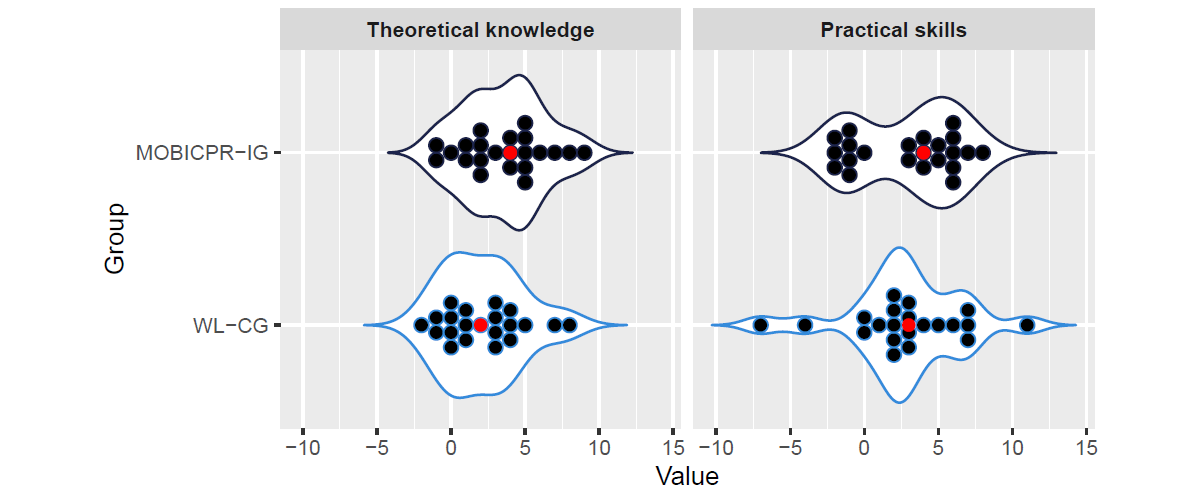
Difference in theoretical knowledge and practical skills score before and after playing the MOBICPR game. MOBICPR-IG: MOBICPR intervention group; WL-CG: wait-list control group.

As demonstrated in Figure 4, in the WL-CG, only 3 (14%) participants improved their theoretical knowledge by ≥5 points and only 6 (29%) study participants who achieved this kind of improvement in the adult BLS practical skill score. In contrast, in the MOBICPR-IG, 9 (41%) participants improved their score by at least 5 points in both adult BLS theoretical knowledge and practical skills. The difference in improvement between the MOBICPR-IG and the WL-CG was not significant in practical skills (η^2^=0.021, *P*=.45), while in theoretical knowledge, we observed a statistically significant difference (η^2^=0.268, *P*=.04).

To obtain more detailed insight into the improvements due to playing the MOBICPR game, we observed the differences in item-level scores before and after playing. Table 2 compares the participants’ scores on questions used to test their theoretical knowledge. It is evident that there were notable differences in most items following engagement with the MOBICPR game. Of 33 scores, 13 (39%) decreased during MOBICPR game playing. For example, the score on question 3 (What is the second thing we check in a patient with cardiac arrest?) improved notably after MOBICPR game playing at home (*P*=.001). In contrast, the score on question 16 (You are alone. Will you go for the AED if it is 100 m away?) did not improve after MOBICPR game playing at home (*P*=.103).

**Table 2:** Question-level comparison of the mean scores for adult BLS^a^ theoretical knowledge evaluation for MOBICPR-IG^b^ and WL-CG^c^ before and after MOBICPR game playing at home for 2 weeks (N=43).

Questions for evaluation of adult BLS theoretical knowledge	Score before playing the MOBICPR game at home, mean (SD)	Score after playing the MOBICPR game at home, mean (SD)	Difference (after – before)	*P* value
1. What is the first thing we check when we approach the patient?	2.51 (0.86)	1.72 (0.43)	–0.79	.04
2. On what kind of surface do we perform adult BLS?	2.88 (0.45)	2.09 (0.34)	–0.79	.001
3. What is the second thing we check in a patient with cardiac arrest?	2.14 (0.74)	2.93 (0.58)	0.79	.001
4. How many seconds do we need to assess consciousness?	1.74 (0.54)	1.95 (0.43)	0.21	.05
5. Before we assess breathing or perform CC^d^, do we remove the patient’s clothes?	1.26 (0.44)	1.77 (0.21)	0.51	.001
6. How do we open the airway?	2.95 (0.21)	1.05 (0.15)	–1.91	.001
7. What maneuver do we use to open the airway?	1.02 (0.15)	2.98 (0)	1.95	.001
8. How do we assess breathing?	2.95 (0.3)	1.00 (0)	–1.95	.001
9. How many seconds do we need to assess breathing?	1.51 (0.86)	3.00 (0)	1.49	.001
10. What is the most common breathing in a patient with cardiac arrest?	2.47 (0.83)	1.00 (0.63)	–1.47	.001
11. Who are you calling on the 112 number?	3.16 (0.37)	2.12 (0.32)	–1.05	.001
12. Will calling 911 in Slovenia or Europe reach emergency services?	1.72 (0.45)	3.12 (0.5)	1.40	.001
13. Who dials 112 in the case of cardiac arrest?	2.81 (0.55)	1.58 (0.3)	–1.23	.001
14. What do we need to tell the emergency medical dispatcher?	2.51 (0.51)	2.95 (0.51)	0.44	.001
15. What do you do with the phone after providing all the data?	1.91 (0.29)	2.70 (0.15)	0.79	.001
16. You are alone. Will you go for the AED^e^ if it is 100 m away?	1.88 (0.32)	1.98 (0.35)	0.09	.10
17. You have help. Will you send it for the AED if it is 2 minutes away?	1.02 (0.15)	1.86 (0.15)	0.84	.001
18. Is this the sign for an AED?	2.00 (0)	1.02 (0)	–0.98	.001
19. Which picture shows the correct hand grip for CPR^f^?	2.00 (0)	2.00 (0.15)	0	.99
20. What is the right depth for CCs?	2.44 (0.77)	2.02 (0.35)	–0.42	.001
21. What is the correct body position for CCs?	1.98 (0.15)	2.86 (0)	0.88	.001
22. Where is the right place for CCs?	1.19 (0.59)	2.00 (0.78)	0.81	.001
23. What is the right frequency for CCs?	2.65 (1.41)	1.23 (0.48)	–1.42	.001
24. What is the CC-to-breath ratio for an adult?	1.98 (0.15)	1.09 (0.46)	–0.88	.001
25. How long can you interrupt CCs for rescue breaths?	1.30 (0.6)	1.93 (0.51)	0.63	.001
26. What is the volume of a rescue breath?	1.88 (0.59)	1.30 (0.26)	–0.58	.02
27. What do you do first if you have an AED?	2.58 (0.91)	2.93 (0.82)	0.35	.002
28. What do we do during AED rhythm analysis?	2.19 (0.59)	2.42 (0.46)	0.23	.17
29. What do we do during AED defibrillation?	1.00 (0)	2.07 (0)	1.07	.001
30. Which of the following statements about the use of AEDs is false?	3.02 (1.3)	1.00 (1.05)	–2.02	.001
31. What do we do after the AED delivers an electric shock?	2.63 (0.69)	3.47 (0.65)	0.84	.001
32. When do we stop CPR?	1.74 (0.44)	2.91 (0.39)	1.16	.001
33. When is it recommended to replace someone during CPR?	1.72 (2.20)	1.81 (0.52)	0.09	.80
Cumulative score	2.08 (0.44)	2.06 (0.26)	0.03	.89

^a^BLS: basic life support.

^b^MOBICPR-IG: MOBICPR intervention group.

^c^WL-CG: wait-list control group.

^d^CC: chest compression.

^e^AED: automated external defibrillator.

^f^CPR: cardiopulmonary resuscitation.

Similarly, in the item-level score differences for practical skills, in 7 (21%) of 34 items, a significant increase was calculated (Table 3). For example, the score on item 1 (Approaches the patient safely) improved after MOBICPR game playing at home (*P*=.001). In contrast, the score on item 2 (Checks responsiveness: shouts and shakes the patient) did not improve after MOBICPR game playing at home (*P*=.81).

**Table 3:** Item-level comparison of the mean scores for adult BLS^a^ practical skill evaluation for MOBICPR-IG^b^ and WL-CG^c^ before and after MOBICPR game playing at home for 2 weeks (N=43).

Items for evaluation of adult BLS practical skills	Score before playing the MOBICPR game at home, mean (SD)	Score after playing the MOBICPR game at home, mean (SD)	Difference (after – before)	*P* value
1. Approaches the patient safely	0.09 (0.29)	0.95 (0.46)	0.86	.001
2. Checks responsiveness: shouts and shakes the patient	0.67 (0.47)	0.70 (0.32)	0.02	.81
3. Opens the airway: head tilt–chin lift	0.40 (0.49)	0.88 (0.46)	0.49	.001
4. Performs look, listen, feel	0.74 (0.44)	0.70 (0.15)	–0.05	.62
5. Looks, listens, feels: time	0.53 (0.74)	0.98 (0.78)	0.44	.001
6. Calls 112 in the first minute	0.65 (0.48)	1.35 (0.26)	0.70	.001
7. Calls 112 at the right time	0.65 (0.48)	0.93 (0.29)	0.28	.003
8. Turns on the phone speaker and immediately starts CPR^d^	0.33 (0.47)	0.91 (0.49)	0.58	.001
9. Provides correct information to the dispatcher	0.33 (0.47)	0.37 (0.5)	0.05	.62
10. Provides information about the location	0.21 (0.41)	0.42 (0.5)	0.21	.05
11. Time to the first CCs^e^	0.51 (0.51)	0.56 (0.5)	0.05	.68
12. Corrects the body position for CCs	0.72 (0.45)	0.53 (0.32)	–0.19	.07
13. Corrects the CC location	0.88 (0.32)	0.88 (0.35)	0.00	.99
14. Corrects hand CCs	0.72 (0.45)	0.86 (0.41)	0.14	.11
15. Corrects the CC depth	1.49 (0.8)	0.79 (0.9)	–0.70	.001
16. Recoil of the chest	0.79 (0.41)	1.26 (0.21)	0.47	.002
17. Corrects the CC rate	1.42 (0.7)	0.95 (0.76)	–0.47	.001
18. Ratios CCs	0.91 (0.68)	1.37 (0.53)	0.47	.004
19. CC fraction	0.84 (0.43)	0.95 (0.38)	0.12	.17
20. Opens the airway: head tilt–chin lift	0.44 (0.5)	0.95 (0.45)	0.51	.001
21. Closes the nose and fits lips around the patient’s mouth	0.63 (0.49)	0.72 (0.37)	0.09	.32
22. Average pause of ventilation	0.70 (0.46)	0.84 (0.43)	0.14	.08
23. Opens the nose	0.02 (0.15)	0.77 (0.32)	0.74	.001
24. Looks for the chest to rise between 2 rescue breaths	0.37 (0.49)	0.12 (0.51)	–0.26	.003
25. Two rescue breaths	0.84 (0.37)	0.51 (0)	–0.33	.001
26. Volume of rescue breaths	0.53 (0.7)	1.00 (0.65)	0.47	.001
27. Switches on the AED^f^ first at the right time	0.49 (0.51)	0.65 (0.46)	0.16	.20
28. Removes clothing	0.98 (0.15)	0.70 (0)	–0.28	.001
29. Position of the right AED pad	0.28 (0.45)	1.00 (0.45)	0.72	.001
30. Position of the left AED pad	0.47 (0.5)	0.28 (0.5)	–0.19	.103
31. Ensures nobody is touching the patient: analyzing	0.51 (0.51)	0.44 (0.5)	–0.07	.58
32. Ensures nobody is touching the patient: shock	0.09 (0.29)	0.58 (0.26)	0.49	.001
33. Presses the shock button at the right time	0.67 (0.47)	0.07 (0.44)	–0.60	.001
34. Immediately restarts CCs	0.95 (0.21)	0.74 (0.29)	–0.21	.002
Cumulative score	0.613 (0.14)	0.76 (0.17)	0.14	.04

^a^BLS: basic life support.

^b^MOBICPR-IG: MOBICPR intervention group.

^c^WL-CG: wait-list control group.

^d^CPR: cardiopulmonary resuscitation.

^e^CC: chest compression.

^f^AED: automated external defibrillator.

### Secondary Outcomes

Table 4 shows a comparison of the high-quality CPR components between participants before and after MOBICPR game playing at home for 2 weeks. There were notable differences in the median (IQR) of the total QCPR score for MOBICPR game playing at home for 2 weeks for the MOBICPR-IG (before: median 41 (IQR 54); after: median 70 (IQR 41); *P*=.011). There was no difference for the MOBICPR-IG after not playing the MOBICPR game at home for 2 weeks.

**Table 4:** Results for high-quality CPR^a^ components for the MOBICPR-IG^b^ and the WL-CG^c^.

High-quality CPR components			Baseline assessment^d^, median (IQR)		Score after 2 weeks of playing the MOBICPR game at home^e^, median (IQR)		*P* value^d,e^		Score after 2 weeks of not playing the MOBICPR game at home^c^, median (IQR)		*P* value^e,f^
**CC^g^ rate (bpm^h^)**											
	MOBICPR-IG	108 (18)		112 (18)		.24		112 (18)		.12	
	WL-CG	103 (22)		110 (10)		.38		—^i^		—	
	*P* value	.78		—		—		—		—	
**CC depth (mm)**											
	MOBICPR-IG	57 (7)		56 (7)		.56		57 (4)		.25	
	WL-CG	58 (6)		59 (2)		.27		—		—	
	*P* value	.16		—		—		—		—	
**CC fraction (%)**											
	MOBICPR-IG	70 (6)		72 (6)		.26		68 (9)		.15	
	WL-CG	68 (13)		70 (8)		.63		—		—	
	*P* value	.88		—		—		—		—	
**Volume of rescue breaths (mL)**											
	MOBICPR-IG	496 (369)		600 (463)		.54		473 (204)		.66	
	WL-CG	356 (147)		567 (270)		.45		—		—	
	*P* value	.64		—		—		—		—	
**Total QCPR^j^ score (%)**											
	MOBICPR-IG	41 (54)		70 (41)		.01		77 (38)		.54	
	WL-CG	43 (42)		72 (46)		.24		—		—	
	*P* value	.62		—		—		—		—	

^a^CPR: cardiopulmonary resuscitation.

^b^MOBICPR-IG: MOBICPR intervention group.

^c^WL-CG: wait-list control group.

^d^Measurement at baseline.

^e^Measurement after 2 weeks of playing the MOBICPR game at home.

^f^Measurement after 2 weeks of not playing the MOBICPR game at home.

^g^CC: chest compression.

^h^bpm: beats per minute.

^i^Not applicable.

^j^QCPR: Quality Cardiopulmonary Resuscitation.

## Discussion

### Principal Findings

In this study, playing the MOBICPR game at home for 2 weeks improved the theoretical knowledge of adult BLS in the participants but little their practical skills. These outcomes were expected, considering that the MOBICPR game was designed primarily to impart theoretical knowledge of adult BLS, rather than providing hands-on practice with an actual BLS manikin. To the best of our knowledge, only 2 studies have used data collected from manikin software to evaluate the practical parts of adult BLS as we did [27,28]. We observed in our study population that both the CC rate and the CC depth remain within the margins of the current ERC recommendation [34]; in comparison to our results, in the 2 studies [27,28], both the CC rate and the CC depth dropped below the margins after serious smartphone game playing. These 2 studies [27,28] also presented the total QCPR scores, and where our scores improved compared to theirs. Consequently, we recommend considering the MOBICPR game as a supplementary educational tool in future BLS course formats that incorporate immersive technologies [43,44] for retention of adult BLS knowledge.

In evaluating study participants performing adult BLS on a manikin, we observed 5 learning points (all reported in Tables 2 and 3), which could be useful for debriefing topics after BLS courses. Initially, a large number of participants struggled with checking the manikin’s response as it lay face down. Some checked the response without turning the manikin onto its back, while others did so with the manikin still face down. After playing the MOBICPR game at home, only a minority checked the response after turning the manikin onto its back. Studies show that two-thirds of all patients are found in positions unsuitable for performing CCs, such as the recovery position [45]. The second learning point concerned the right time for chest exposure during CPR. Many participants removed the clothing before looking, listening, and feeling for signs of breathing, while others did so before applying AED electrodes to the manikin’s bare chest. Studies indicate that exposing the chest during CPR can improve the rescuer’s ability to locate the center of the patient’s chest, leading to more effective CCs and reducing the risk of inaccurate compressions [46]. The third point was about shouting for help. A recent study revealed that almost all European BLS instructors teach laypersons to shout for help [47], even though it was removed from the ERC BLS guidelines [34,40]. Despite playing the MOBICPR game at home, the participants still tended to shout for help before calling emergency services. As a fourth point, we noticed that some participants attempted to multitask by calling the dispatcher and performing CCs simultaneously. This practice resulted in lower-quality CCs, as the focus was divided between providing information to the dispatcher and maintaining the 30:2 CC-to-rescue-breath ratio. Generation Z, like the participants in our study, tends to multitask and is more engaged in independent work [48]. Considering this insight, we are re-evaluating the recent ERC BLS guidelines, particularly their recommendation to activate the speakerphone or another hands-free feature on a mobile device before promptly initiating CPR [34]. Finally, we observed that almost all study participants failed to ensure safety before defibrillation when using an AED on the manikin. Issues arose before pressing the shock button, either because they did not check whether someone was touching the manikin or because they pressed the shock button prematurely. This highlights that using an AED is not intuitive for laypersons, as studies suggest, and special training should be considered [49].

The International Liaison Committee on Resuscitation provides a scientific statement on teaching laypersons adult BLS and suggests using TEL, such as serious smartphone games, to engage, motivate, and educate children and adolescents in saving more lives [34]. Several legitimate smartphone games have been identified as suitable for teaching adult BLS, but their content is questionable because it does not follow current BLS guidelines [50,51]. Moreover, most of them teach only hands-on CPR. Some also include ventilation and AED use [51]. However, the MOBICPR game was developed based on recent ERC BLS guidelines [34] and includes all the recommended BLS steps. In a recent MOBICPR study, students agreed that it was beneficial to play the MOBICPR game before practicing adult BLS on a manikin [41]. They also highly rated the usability of the MOBICPR game for providing adult BLS theoretical knowledge and practical skills. The results show that the MOBICPR game could be a novel, interactive, evidence-based BLS educational tool for playing at home after adult BLS training [41,52]. Moreover, our study revealed that the MOBICPR game could be an effective method for enhancing bystander willingness and awareness in performing CPR. This potential is demonstrated by the fact that all study participants introduced the MOBICPR game to their family members, relatives, or friends, as seen in similar studies where enhanced technology was used teaching adult BLS [53].

This gamified learning approach fits well with the educational theory heutagogy, also known as self-determined learning, where learners determine what they want to learn [8]. In the case of the MOBICPR game, learners can play it at any time to refresh their adult BLS knowledge without waiting for the next training session [54]. Moreover, the use of do-it-yourself manikins made from everyday items, such as plastic bottles, toilet paper, or even a pillow, for practicing CC techniques at home, especially in low-resource settings, coupled with the MOBICPR game, can potentially improve and solidify practical skills in adult BLS [55-58]. The MOBICPR game also includes gamification features, such as avatars, points, and various audio, textual, and graphical feedback. These gamification elements could motivate learners to engage with the game more frequently than they normally would [59]. Future educational tools, such as the MOBICPR game, should align with the 5 key messages outlined in the recent ERC BLS guidelines, ranging from recognizing cardiac arrest to learning the proper techniques for performing CPR [34]. This adherence is crucial for the effective education and retention of adult BLS skills, particularly following adult BLS courses in a home environment.

### Limitations

This study has several limitations. First, because the study participants were only followed for 4 weeks, we were not able to show that the MOBICPR game improved their long-term retention of resuscitation knowledge and skills. Second, the sample size was small due to the lack of interest of participants in participating in the study and because only 1 generation of participants was able to be included at that time. Third, this was a single-faculty study, which limits the generalizability of the results. Fourth, in this study, participants were familiar with smartphone games. It is unclear how effective the MOBICPR game would be in children or older populations. Fifth, because this was a simulation-based study, the performance results may not be generalizable to real-life situations and could not present the impact on patient outcomes. Finaly, the content in the MOBICPR game was developed by researchers based on recent ERC BLS guidelines [34]. In the future, there are plans to introduce the MOBICPR game to the Slovenian National Resuscitation Council, with the goal of securing its certification, a process akin to that followed by the Italian Resuscitation Council for its smartphone-based serious games [60].

### Conclusion

The home use of the MOBICPR game shows promise in enhancing the theoretical knowledge of adult BLS. Although there was no significant improvement in performing adult BLS or in retaining the related knowledge and skills, the study yielded important learning objectives for the enhancement of future adult BLS training. Further research is necessary to explore its lasting effects across various demographics and to determine the most effective use of the MOBICPR game in teaching adult BLS.

## Appendix

Multimedia Appendix 1 CONSORT-eHEALTH checklist (V 1.6.1).

Multimedia Appendix 2 Adult BLS theoretical knowledge questionnaire. BLS: basic life support.

Multimedia Appendix 3 Out-of-hospital cardiac arrest scenario.

Multimedia Appendix 4 Adult BLS practical skills checklist. BLS: basic life support.

## Abbreviations

AED: automated external defibrillator
BLS: basic life support
bpm: beats per minute
CC: chest compression
CPR: cardiopulmonary resuscitation
ERC: European Resuscitation Council
m-learning: mobile learning
MOBICPR-IG: MOBICPR intervention group
OHCA: out-of-hospital cardiac arrest
QCPR: Quality Cardiopulmonary Resuscitation
TEL: technology-enhanced learning
WL-CG: wait-list control group
