# Supplementary material for: Effects of a Serious Smartphone Game on Nursing Students' Theoretical Knowledge and Practical Skills in Adult Basic Life Support: Randomized Wait List–Controlled Trial
Source: JMIR Serious Games. 2024 Apr 5;12:e56037. doi: 10.2196/56037 (PMC11031703; doi:10.2196/56037)
Supplement: Multimedia Appendix 2 [file games_v12i1e56037_app2.docx]

**Appendix 2:** Adult BLS theoretical knowledge questionnaire

| **Questions and answers** | **Points** |
| --- | --- |
| 1. **What is the first thing we check when we approach the victim?** 2. Pulse. 3. Safety. 4. Breathing. 5. Consciousness. | 0  1  0  0 |
| 1. **On what kind of surface do we perform adult BLS?** 2. On a medium soft surface and when a person is lying on their back. 3. On hard, uneven surface and when a person is lying on their stomach. 4. On a hard, level surface and with a person lying on their back. | 0  0  1 |
| 1. **What is the second thing we check in a cardiac arrest victim?** 2. Shake the person strongly to try to wake them up and wait for help. 3. Gently shake the person's shoulders and ask out loud, "Are you OK?" to see if the person is responding. 4. Immediately start with chest compressions and rescue breaths. 5. Send someone to get an automated external defibrillator. | 0  1  0  0 |
| 1. **How many seconds do we need to assess consciousness?** 2. 5 seconds. 3. 10 seconds. 4. 20 seconds. | 1  0  0 |
| 1. **Before we assess breathing or perform chest compressions, we remove clothes?** 2. True. 3. False. | 1  0 |
| 1. **How do we open the airway?** 2. We use force to remove dental prostheses. 3. We don't remove anything. 4. We remove all visible foreign objects from the mouth, including the dentures if it is not well attached. 5. We tap the person lightly on the back. | 0  0  1  0 |
| 1. **What manoeuvre do we use to open the airway?** 2. We tilt the head back and lift the chin. 3. We lift the lower jaw. 4. We lift the chin.. | 1  0  0 |
| 1. **How do we assess breathing?** 2. We do a pulse check on the neck. 3. We ask a colleague if they notice anything. 4. We look towards the chest, listen and feel for the breathing. 5. We record it with a smartphone. | 0  0  1  0 |
| 1. **How many seconds do we need to assess breathing?** 2. 10 seconds. 3. 20 seconds. 4. 5 seconds. | 1  0  0 |
| 1. **What is the most common breathing in cardiac arrest victims?**   (https://univerzamb-my.sharepoint.com/:u:/g/personal/nino_fijacko_um_si/EQlshrm8fZRFh5Qgr8SDQ0UBXxf2m0mr7S7TLtOHaBph0A?e=zSFFHe)   1. Stridor 2. Agonal breathing. 3. Choking 4. Apnoea. | 0  1  0  0 |
| 1. **Who are you calling on the 112 number?** 2. Police department. 3. Fire department. 4. 112 Center (emergency medical services, firefighters). 5. Emergency medical services. | 0  0  1  0 |
| 1. **Calling 911 in Slovenia or Europe will reach emergency services?** 2. True. 3. False. | 1  0 |
| 1. **Who dials 112 in case of cardiac arrest?** 2. I do. 3. Other witnesses. 4. Both answers are correct. | 0  0  1 |
| 1. **What do we need to tell the emergency medical dispatcher?** 2. What, where and when it happened, what are the injuries. 3. What, where and when it happened, the number of victims and the nature of the injuries. 4. Who is calling, what, where, when it happened, the number of victims, the nature of their injuries and your telephone number. 5. We do not need to provide any information as they will find us via GPS. | 0  0  1  0 |
| 1. **What do you do with the phone, after all the given data?** 2. Turn off your smartphone and put it in your pocket. 3. Put your smartphone on speakerphone or other hands-free mode and place it near the victim's head. | 0  1 |
| 1. **You are alone. Will you go for the AED if it is 100 m away?** 2. Yes. 3. No. | 0  1 |
| 1. **You have help. Will you send it for the AED if it is 2 min away?** 2. Yes. 3. No. | 1  0 |
| 1. **Is this the sign for AED?**   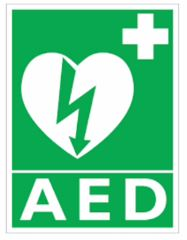   1. No. 2. Yes. | 0  1 |
| 1. **Which picture shows the correct hand grip for CPR?** 2. 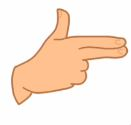 3. 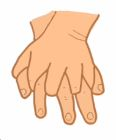 4. 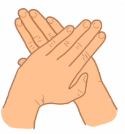 | 0  1  0 |
| 1. **What is the right depth for chest compressions?** 2. 7-8 cm or the length of a middle finger. 3. 6-7 cm or the length of an index finger. 4. 5-6 cm or the length of a thumb. | 0  0  1 |
| 1. **What is the correct body position for chest compressions?** 2. 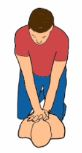 3. 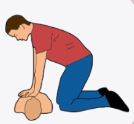 | 0  1 |
| 1. **Where is the right place for chest compressions?** 2. The lower half of the sternum and in the middle of the chest. 3. The lower half of the sternum and on left side of the chest. 4. The lower half of the sternum and on right side of the chest. 5. Straight on the heart. | 1  0  0  0 |
| 1. **What is the right frequency for chest compressions?** 2. At least 100 chest compressions per minute. 3. At least 150 chest compressions per minute. 4. At least 80 chest compressions per minute. 5. At least 60 chest compressions per minute. | 1  0  0  0 |
| 1. **What is the chest compression to breath ratio for an adult?** 2. 15 (chest compressions): 2 (breaths). 3. 30 (chest compressions): 2 (breaths). 4. 30 (chest compressions): 5 (breaths). 5. 15 (chest compressions): 1 (breaths). | 0  1  0  0 |
| 1. **How long can you interrupt chest compressions for rescue breaths?** 2. 10 seconds. 3. 20 seconds. 4. 30 seconds. | 1  0  0 |
| 1. **What is the volume of rescue breath?** 2. 300–400 ml. 3. 400–500 ml. 4. 500–600 ml. | 0  0  1 |
| 1. **What do you do first if you have an AED?** 2. Stop CPR immediately. 3. Turn on the AED and follow the instructions. 4. Open the electrodes and connect them to the AED. 5. Continue CPR until a cycle of 30:2 is completed before using an AED. | 0  1  0  0 |
| 1. **What do we do during AED rhythm analysis?** 2. We hold the person's shoulder. 3. We do not touch the victim. 4. We turn off the AED. 5. We check the pulse. | 0  1  0  0 |
| 1. **What do we do during AED defibrillation?** 2. We all move away from the victim /do not touch them. 3. We ask the person how he or she feels. 4. We hold the vistim's hand. 5. We regularly check the pulse and breathing. | 1  0  0  0 |
| 1. **Which of the following statements about the use of AEDs is false?** 2. An electric shock can be delivered if a victim lies on a wet floor. 3. An electric shock can be delivered if the person has a pacemaker. 4. CPR can continue while the AED analyses the victim's heart rhythm. 5. All of the above. | 1  0  0  0 |
| 1. **What do we do after the AED delivers an electric shock?** 2. Place the person in the recovery position. 3. Check the pulse. 4. We start with CPR with 30:2 ratio. 5. Check breathing. | 0  0  1  0 |
| 1. **When do we stop CPR?** 2. When the person shows definite signs of life and when the emergency services arrive. 3. If the family members say that it is no longer necessary. 4. When we hear the siren of the emergency vehicle. 5. When we are exhausted and when we are replaced by another person. | 1  0  0  1 |
| 1. **When is it recommended to replace someone during CPR?** 2. After 1 minute. 3. After 2 minutes. 4. After 4 minutes. 5. After 6 minutes. | 0  1  0  0 |
| **Total number score:** | **33 points** |
