# Supplementary material for: Effects of a Serious Smartphone Game on Nursing Students' Theoretical Knowledge and Practical Skills in Adult Basic Life Support: Randomized Wait List–Controlled Trial
Source: JMIR Serious Games. 2024 Apr 5;12:e56037. doi: 10.2196/56037 (PMC11031703; doi:10.2196/56037)
Supplement: Multimedia Appendix 3 [file games_v12i1e56037_app3.docx]

**Appendix 3:** Out-of-hospital cardiac arrest scenario

You are called to help an adult who is lying on the floor. This person appears to be your mother or father. Emergency services have not been notified. There is an automated external defibrillator in the building. You have an assistant who will be available in approximately 2-3 minutes to provide an automated external defibrillator (AED) and replace you in cardiopulmonary resuscitation. In the meantime, please continue with adult basic life support on your own. We will be on hand to answer questions (for example, if you ask: "Is the victim breathing?", expect a "Yes" or "No" answer). The scenario will last approximately 10 minutes.
