# Supplementary material for: Effects of a Serious Smartphone Game on Nursing Students' Theoretical Knowledge and Practical Skills in Adult Basic Life Support: Randomized Wait List–Controlled Trial
Source: JMIR Serious Games. 2024 Apr 5;12:e56037. doi: 10.2196/56037 (PMC11031703; doi:10.2196/56037)
Supplement: Multimedia Appendix 4 [file games_v12i1e56037_app4.docx]

**Appendix 4:** Adult BLS practical skills checklist

| **Adult BLS checklist** | **Points** |
| --- | --- |
| 1. **Approaches the victim safely:** 2. Not performed 3. Performed | 0  1 |
| 1. **Check responsiveness: shout and shake:** 2. Not performed 3. Performed | 0  1 |
| 1. **Open airway: head tilt-chin lift:** 2. Not performed 3. Performed | 0  1 |
| 1. **Look, listen, feel (position head close to the victims face, watch into victim chest):** 2. Not performed 3. Performed | 0  1 |
| 1. **Look, listen, feel: Time:** 2. 1-3 3. 4-7 4. 8-12 | 0  1 2 |
| 1. **Call 112 in the first minute:** 2. More than 60 s 3. Less than 60 s | 0  1 |
| 1. **Call 112 at the right time (e.g. before CC):** 2. Not performed 3. Performed 4. Performed but first start with CPR | 0  1  0 |
| 1. **Turn on the speaker function on the phone and immediately start CC:** 2. Not performed 3. Performed | 0  1 |
| 1. **Provide information that the victim is unresponsive with absent/abnormal breathing or cardiac arrest:** 2. Not performed 3. Performed | 0  1 |
| 1. **Provide information of the OHCA location:** 2. Not performed 3. Performed | 0  1 |
| 1. **Time to first CC:** 2. More than 30 s 3. Less than 30 s | 0  1 |
| 1. **Correct body position for CC (kneel, position, arms straight):** 2. No 3. Yes | 0  1 |
| 1. **Correct CC location (the lower half of the victim's breastbone/sternum):** 2. No 3. Yes | 0  1 |
| 1. **Correct hand CC (hand on hand, interlock your fingers):** 2. No 3. Yes | 0  1 |
| 1. **CC depth:** 2. Less than 4.0 cm 3. 4.1–4.9 4. 5.0–6.0 5. 6.0–6.5 6. More than 6.5 | 0 1 2 1 0 |
| 1. **CC recoil:** 2. No 3. Yes | 0  1 |
| 1. **CC rate:** 2. 60–79 3. 80–99 4. 100–120 5. 121–129 6. More than 130 | 0  1  2  1  0 |
| 1. **CC ratio:** 2. 25–27 3. 28–32 4. 33–35 | 1  2  1 |
| 1. **CC fraction:** 2. Less than 60% 3. 60–80% 4. More than 80% | 0  1  2 |
| 1. **Open the airway again, using head tilt and chin lift:** 2. No 3. Yes | 0  1 |
| 1. **Two rescue breaths:** 2. No 3. Yes | 0  1 |
| 1. **Closed the nose and fits lips around victims mount:** 2. No 3. Yes | 0 1 |
| 1. **Average ventilation pause:** 2. 0 s 3. 0.1–1.0 s | 0 1 |
| 1. **Open the nose and remove lips around victims mount:** 2. No 3. Yes | 0 1 |
| 1. **Look chest to rise beetwen 2 rescue breaths:** 2. No 3. Yes | 0 1 |
| 1. **Average volumen of rescue breath:** 2. 1–299 ml 3. 300–499 ml 4. 500–600 ml 5. 601–799 ml 6. More than 800 ml | 0  1  2  1  0 |
| 1. **Switch on AED first at the right time:** 2. No 3. Yes | 0 1 |
| 1. **Remove clothing:** 2. No 3. Yes | 0 1 |
| 1. **Correct right AED pad:** 2. No 3. Yes | 0 1 |
| 1. **Correct left AED pad:** 2. No 3. Yes | 0 1 |
| 1. **Ensure nobody is touching the victim 1: Analysing:** 2. No 3. Yes | 0 1 |
| 1. **Ensure nobody is touching the victim 2: Shock** 2. No 3. Yes (voice, look, gesture) | 0 1 |
| 1. **Press the shock button at the right time:** 2. No 3. Yes | 0 1 |
| 1. **Immediately restart CC:** 2. More than 5 s 3. Less than 5 s | 0 1 |
| **Total number score:** | **39 points** |
